# The Verification of Nanosatellites Solar Panels Automatic Deployment in Microgravity Conditions

**DOI:** 10.1007/s42423-021-00083-4

**Published:** 2021-08-12

**Authors:** Akram Abdellatif, Ali H. Ali, Mohamed E. El-sayed, Nermine M. Elhusseiny, Youmna Mabrouk, Youssef M. Fathy

**Affiliations:** 1 Flight Experiments, German Aerospace Center (DLR), Wessling, Germany; 2 German University in Cairo (GUC), Cairo, Egypt

**Keywords:** CubeSat, Structural analysis, Nanosatellite, Microgravity experiment, Solar power analysis

## Abstract

The solar panels installed on a CubeSat are considered the main energy source of a nanosatellites. The deployment mechanism of a solar panel must be analyzed and tested extensively. Any suggested solar panel design should present a low vibrating free spinning deployment mechanism. This paper examines various types of solar panels to reach a conclusion of the efficient design when deployed on a 1U or 2U unit. However, calculations, analysis, simulations do not always give an extensive picture of how the satellite shall behave during deployment. Thus, testing in a microgravity environment gives a more accurate answer of how the satellite shall behave. In our work, various solar panels mechanisms are developed and eventually tested in microgravity. The first accordion structure for a 1U structure is tested in a microgravity environment through a parabolic flight with the National Research Council Falcon 20 aircraft. The results are recorded and analyzed to optimize the next design. The second design is based on a drag-sail mechanism for a 2U structure. The design is improved upon the first experiment results for the next parabolic flight. The simulated amount of power generated in orbit is also a main factor in our evaluation.

## Introduction

The CubeSat project was launched by the University of Stanford and Cali Poly in 1999 to provide a pico-satellite standard with a lower cost and development time, increase space accessibility, and maintain frequent launches. The 1U CubeSat is a 10-cm cube up to 1.33 kg in weight [1]. Several variations like 2U, 3U, and 6U have been applied to the form factor. Although this standardization has many benefits for the designers of these systems, there are drawbacks to the inherent limitation of volume, for example for payloads requiring large solar generators. Deployable and expandable systems are a versatile way of overcoming limitations and of enabling advanced payloads on CubeSat. Examples of this include deployable antennas, solar sails, and solar panel arrays. In these cases, a primary advantage is that large deployment ratios or large deployed-to-stowed size ratios can be obtained. Higher deployment ratios are significant for CubeSats, because they increase the mass efficiency of the deployable. They have a lower launch volume and mass; however, once in space the system unfolds to provide a large surface area, it is perfect for power generation and transmission. The dual usage of this surface is very advantageous.

In this paper, two expandable designs were proposed to increase the number of additional solar panels on the 1U and 2U CubeSats and accordingly increase power generation. For the 1U CubeSat, an accordion origami concept is implemented, while for the 2U CubeSat, the drag-sail mechanism is used. Different tests and analyses are done on both designs to prove their practicability, such as structural analysis, dynamical analysis, power analysis, and microgravity experiment using a parabolic flight.

In Sect. 2, related work on the CubeSats are discussed. Section 3 contains the mechanical designs of both the 1U and 2U CubeSats. The motion analysis is discussed in Sect. 4, while the power analysis is discussed in Sect. 5. Section 6 covers the microgravity experiment on the 1U CubeSat and the parabolic flight safety constraints. Finally, conclusion and future work are present in Sect. 7.

## Related Work

The mechanical component concerns CubeSat’s durability and strength as it is deployed into the outside space. During the process, the CubeSat mainly works in three forms of mechanical load: quasi-static, static, and dynamic loading. The work in [2] executed a static loading analysis acting on a CubeSat. They concern about stress distribution and deformation due to the static loading of the chassis of CubeSat. The CubeSat were model as 10 $\times$ 10 $\times$ 10 cm (1U). ANSYS.19 was used for static analysis. The results show that by increasing the number of holes in the structure, the total deformation increases. It also showed that the edge of the structure has the highest stresses.

The work represented in [3] includes design and analysis of a remote-sensing satellite structure. The content of the paper focused on the selection of material and optimization of the geometry of the model. The center of mass of the structure had to be within the range of 1–2 cm from the center of the geometry. The model was analyzed statically using ANSYS software under specific loading and boundary conditions to ensure that the structure keeps its integrity during launching. A model of mono block was introduced as the structure to be analyzed using Aluminum alloy 7075-T6. The static analysis performed was used to estimate stresses, forces, strains, and displacements in the components of the structure to measure the strength of the satellite model. The forces in this analysis were the weight of the satellite and a 9 g force that were acted on the geometric center of the model and fixed supports were used for the lower legs of the base of the structure. Results showed that the maximum stresses and deformation were found at the top sheet. The maximum equivalent stress was lower than the yield strength of Aluminum alloy 7075-T6 and the maximum deformation was far less compared to the dimensions of the structure. Therefore, the structure was proved to bear the loading conditions, maintaining its integrity, and does not fail during launching [3].

In [4], the work presents the finite-element analysis for the 3U CubeSat structure. The CubeSat must not fail when encounter the highest static and dynamic loads during launching and its lifespan. Therefore, they ensured that it will not face unacceptable stresses, deformation, strain, and displacements during the launch by making a static structural analysis using ANSYS software to test CubeSat design using computer-based model before machining the actual CubeSat to provide a low-cost testing solution, optimize bodies, and enhance the performance to meet the requirements in the intended environment. They chose Aluminum 7075-T6 as the structural material for their design model in ANSYS, because it has high yield strength, low cost, and lightweight and can be machined easily. They applied up to 50 g force on the center of the geometry and fixed the lower legs of the base. The results of the static analysis showed that the maximum equivalent stress was lower than the yield strength of Aluminum 7075-T6 and the maximum deformation was far less as compared to the dimensions of the structure.

The static response of their primary and secondary structure which is done using ANSYS software was shown in [5]. This response was performed to be compared with the strength requirements to make sure that the structure will not fail during launching. Aluminum 6061-T6 was chosen for the structure because of its relatively high strength, high resistance to corrosion, and excellent joining characteristics. The maximum quasi-static launch load was applied in the three flight directions which are *x*-, *y*-, and *z*- separately. Results of equivalent stress (Von-Mises) distributions showed that the maximum stress that the primary and secondary structures experience is safe as it was small compared to the yield stress of Aluminum 6061-T6 and the total deflection was acceptable in comparison to the static deflection envelopes of the all components of the structures.

NASA’s Jet Propulsion engineers in Pasadena, California, think about how the principles of origami could be used for space-bound devices for easier deployment. Researchers say that origami can be useful in utilizing space solar power for earth-based purposes. Solar power acting like a power planet that wirelessly beams power down to Earth using microwaves. Panels used in space missions needed to be simple folds, collapsing like a fan or an accordion to simplify mechanical design for opening and closing [6].

[7] created a deployable solar sail within a 2U CubeSat frame with a prototype folding using the Geneva mechanism as an easily deployable mechanism which prevents the unwanted rotation while opening and closing.

### Power Analysis

A standard 1U Cubesat is proposed to have increased solar panels to increase the power generated [8]. This modification is done to the 1U cube to be called HeidelSat. Two extra solar panels were added to each side to have a total of eight expandable solar panels added to the cube. Therefore, in the new design, there are an additional 32 solar cells with an average efficiency of 30%. It is designed for low earth orbit (LEO) as the height of the orbit is about 400 km. A simulation is done to check the power generated. This modification of HeidelSat has a minimum power of 4.8 W, maximum power of 12.2 W, and average power of 8.4 W. Therefore, the total generated power increased according to the simulation done.

## Mechanical Design

### Accordion Design

**Fig. 1:**
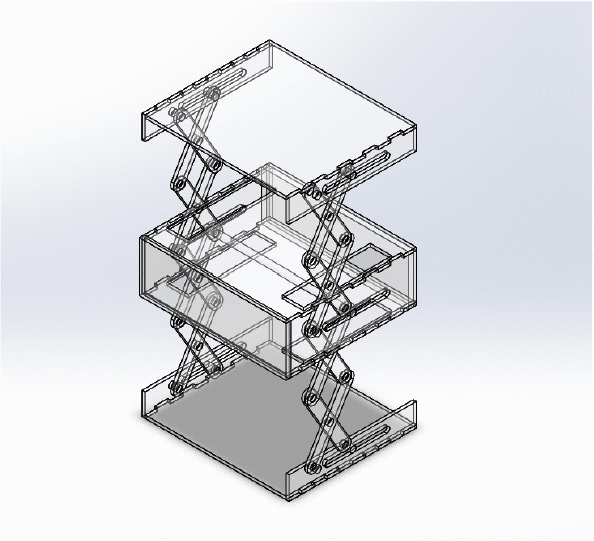
Accordion CubeSat design

The accordion can provide a large surface area from a small one with a simple mechanism. The accordion folds with be used to add the solar panels on it to increase the number of the solar panels of the cube-sat. The mechanism that will fold and unfold the accordion origami will be scissors mechanism which is used in elevators and hydraulic lifts. The accordion extendable CubeSat mechanism which is designed using SOLIDWORKS [9] is shown in Fig. 1. The design consists of two symmetric scissors that are coupled together to guarantee their symmetry. The scissors are opened and closed using transitional motion.

### Drag Sail

Drag sail is another type of origami folding technique that has a unique design to provide a large area of solar panels while opening. Drag-sail design consists of three main parts which are frame design, rotating mounting plate and Geneva cam represents unfold mechanism.

**Fig. 2:**
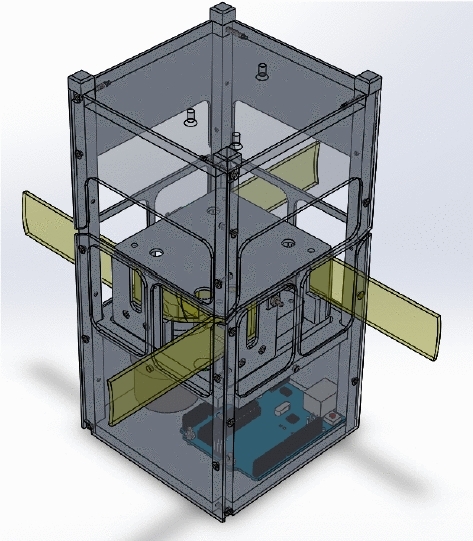
Drag-sail design [7]

**Fig. 3:**
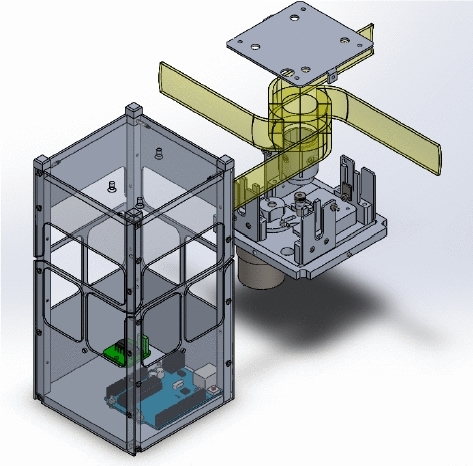
Drag-sail design parts [7]

As shown in Figs. 2 and 3, prototype design has thin sheet frames from 6061 aluminum material also has a gyroscopic mounting plate allows for the implementation of sail orientation. The mechanism used for fold and unfold origami is the Geneva Cam mechanism which provides the extension of four end meter moves to outside cube bodies to unfold origami. This figures explain the drag-sail mechanism without adding the panels . The panels are added in Fig. 19 .

**Fig. 4:**
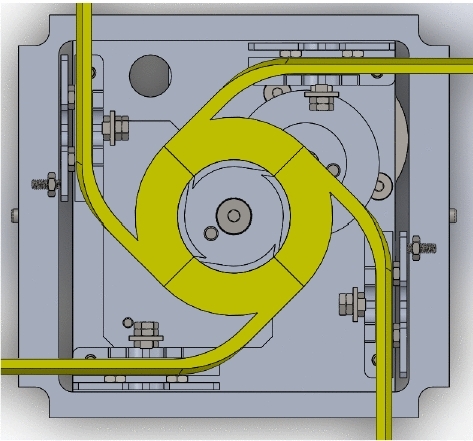
Drag-sail mechanism [7]

**Fig. 5:**
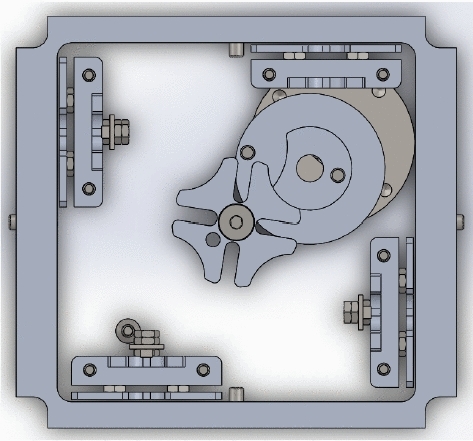
Drag-sail Geneva Cam [7]

As shown in Figs. 4 and 5, the Geneva mechanism used for Drag Sail consists of a drive wheel attached to the motor shaft, pin in area of the drive wheel and driven wheel attached to coupler. The mechanism allows to 4 m to extend or retract in one direction and prevents an unwanted rotation in the mechanism. Coupler attached to Geneva driven wheel has the main role to rotate the 4 end meters in the four guided plates sides the cube body. Extension and retraction of four meters with the same disablement will provide the right fold and unfold for origami sail without slipping or returning because of the Geneva Cam stop duration.

## Motion Analysis

### Accordion Design

**Fig. 6:**
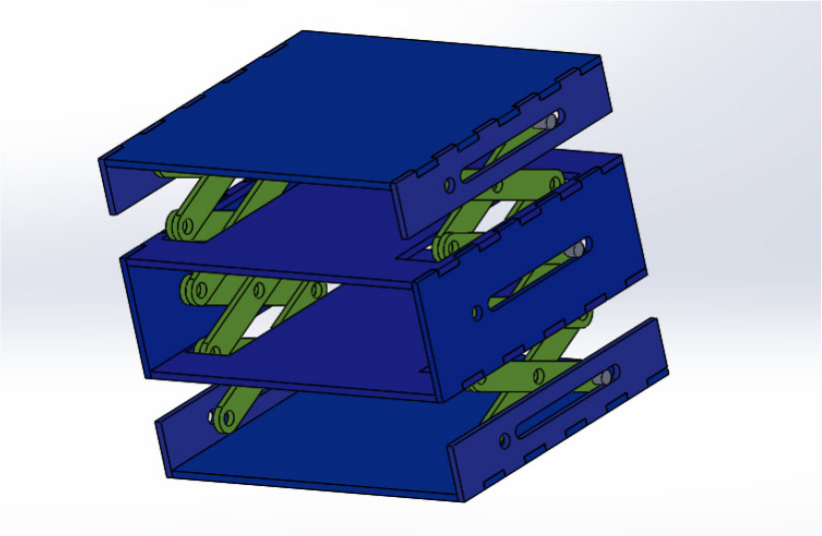
Accordion design at *t* = 0

**Fig. 7:**
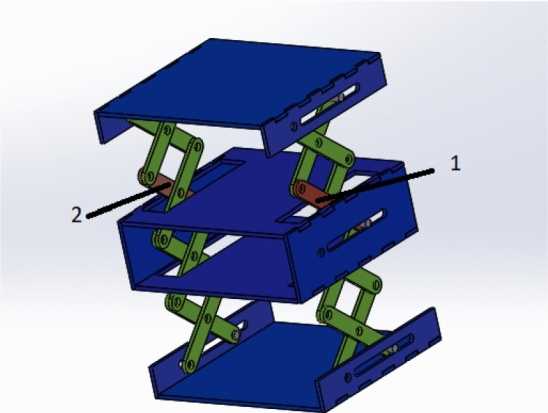
Accordion design at *t* = 1.8 s

During the microgravity envelope, the side forces or extra torques will lead to unstable panels deployment. Therefore, motion analysis is made to the grantee that the mechanisms of the extendable CubeSat designs will not lead to extra forces or torques and the velocities of the joints of the mechanism are symmetric. For the accordion mechanism, SOLIDWORKS motion analysis is used, as shown in Figs. 6 and  7. This analysis converts the design into the finite-element model and results in the reaction forces and velocity of the links and joints.

**Fig. 8:**
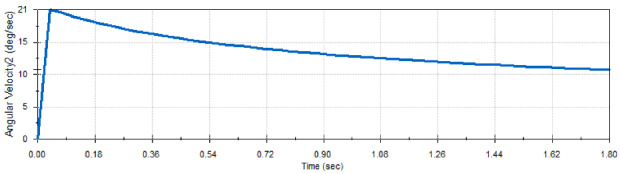
Velocity of link 1

**Fig. 9:**
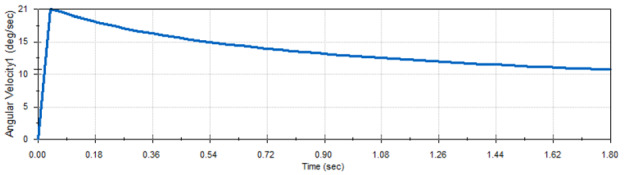
Velocity of link 2

The motion analysis showed that every two corresponding links in the scissor mechanism have the same velocity. Figures 8 and 9 show the velocity of link 1 and link 2 which are mentioned in Fig. 7. This design is simulating the scissors mechanism without adding the panels . The design after adding the panels will be explained in Fig. 16.

**Fig. 10:**
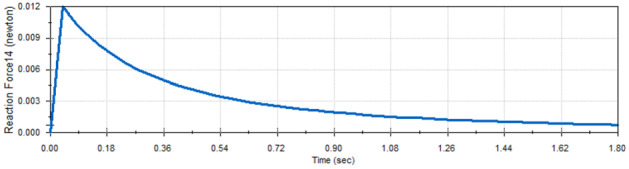
Reaction force of mid-joint of link 1

**Fig. 11:**
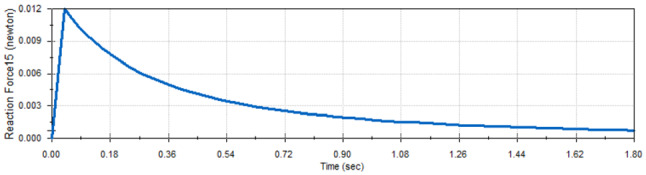
Reaction force of mid-joint of link 2

The analysis also showed that the scissors mechanism guarantees that the reactions of each two corresponding joints are the same which guarantees that there are neither side forces nor extra torques during panels deployment in the microgravity experiment. Figures 10 and 11 show the reaction of the middle joints of link 1 and link 2 which is specified in Fig. 7.

### Drag-Sail Design

#### Motion Analysis for Geneva Mechanism

Drag-sail design should verify that the 4 m move with the same velocity to achieve successful folding for origami sail without any fail on opening.

**Fig. 12:**
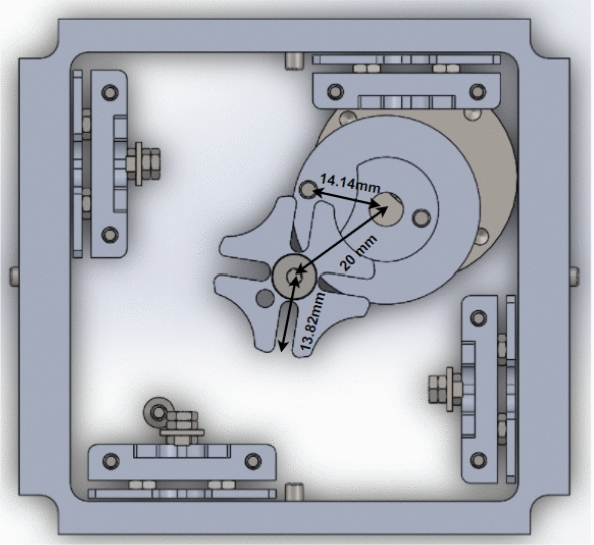
Geneva mechanism

Motion analysis for Geneva mechanism will be proceeded by giving the motor a certain $\omega _\mathrm{{in}}$ with studying output $\omega _\mathrm{{out}}$ through Geneva cam-driven wheel. As shown in Fig. 12, the graph represents the motion of the driven wheel with respect to driver wheel $\theta _\mathrm{w}$. The wheel rotates only in a certain range of full-cycle driver rotation and the rest of cycle in dwell period. Geneva cam stops the driven wheel to return back in range of dwell period to prevent the stored energy of meter to go in another direction. The parameters used for $\omega _\mathrm{{out}}$ analysis calculations are:

- Center-to-center diameter $(d_\mathrm{c}= 20)$ mm
- Wheel diameter $(d_\mathrm{w} = 13.82240677)$ mm.
- Driver pin diameter $(d_\mathrm{p}= 14.14)$ mm.
- $\theta$ is the theta of driver wheel with + *x* axis.
- $\alpha _\mathrm{w}$ the angle of driven wheel.
- $\omega _\mathrm{m}$ the velocity of driver wheel.

Studying $\omega _\mathrm{{out}}$ behavior by changing the angle $\theta$ from $0^\circ$ to $360^\circ$ (full rotation)

1 $$\begin{aligned} d^2= & {} d_\mathrm{p}^2 + d_\mathrm{c}^2 - 2*d_\mathrm{p}*d_\mathrm{c}* \cos (\theta ), \end{aligned}$$

2 $$\begin{aligned} d_\mathrm{p}*\omega _\mathrm{m}*\sin (\theta )= & {} V_\mathrm{p}*\cos (\alpha )- d*\omega _{2}*\sin (\alpha ), \end{aligned}$$

3 $$\begin{aligned} d_\mathrm{p}*\omega _\mathrm{m}*\cos (\theta )= & {} -V_\mathrm{p}*\sin (\alpha ) -d*\omega _{2}*\cos (\alpha ). \end{aligned}$$

**Fig. 13:**
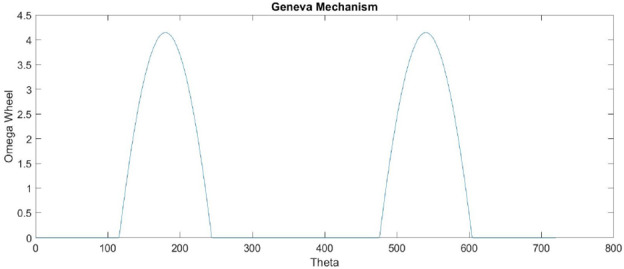
Velocity of Geneva Cam

Also, the mechanism provides the same displacement of 4 m, because 4 m attached pins are connected with coupler with the same diameter from the coupler center. The velocity of the cam will be the same velocity of meter extension because of symmetric meters attached with coupler. As a result of velocity and transmission of the Geneva cam mechanism, the 4 m will provide symmetric velocity and displacement output for extension and retraction process (unfold, fold).

## Power Analysis

To evaluate the CubeSats performance during its presence in the space, the two proposed CubeSats models are imported to Systems Tool Kit (STK) program [10] which is a solar panel simulation tool to measure the generated power. The model is assumed to be in a fully extended state during the complete simulation interval.

### Solar Cells

**Fig. 14:**
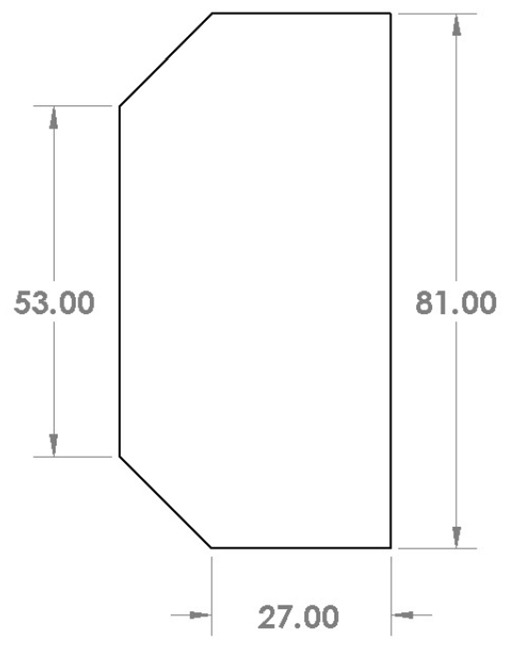
AZUR solar cell’s dimensions

Solar cells, which are placed on the solar panels, are used for power generation. AZUR space solar cells are chosen, which are space proved gallium arsenide solar cells, called GaAs triple-junction solar cells [11]. The type “TJ Solar Cell 3G30C” is a solar cell with an average efficiency of 30%. These solar cells have an integrated by-pass diode for protection of an adjacent cell in a string of solar cells. The area of the cell is about $3125 \mathrm{{mm}}^2$ with a mass less than 2.6 grams. The dimension of the solar cell is shown in Fig. 14. The number of solar cells mounted on each CubeSat is shown in Table 1.

**Table 1:** Number of solar cells based on the type of the satellite

Unit	Solar cells	Efficiency $(\%)$
1U	12	28
Accordion CubeSat	24	30
2U	20	28
Drag-Sail CubeSat	44	30

### Orbit Features

There are four different parameters that define the orbit which is the right ascension of ascending node (RAAN), eccentricity, inclination, and height. The mentioned orbit parameters values are shown in Table 2. These values are used for all the simulations on STK. The International Space Station (ISS) orbit is considered as it is the most likely used orbit for CubeSats.

**Table 2:** Orbit parameters

RAAN	Eccentricity	Inclination	Height
$40 ^\circ$	0	$51.6414 ^\circ$	400 km

### Accordion CubeSat

**Fig. 15:**
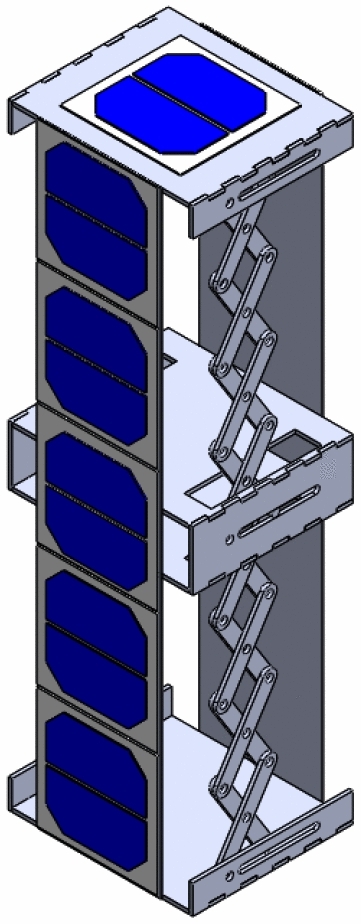
Isometric view of the fully extended 1U Accordion CubeSat

**Fig. 16:**
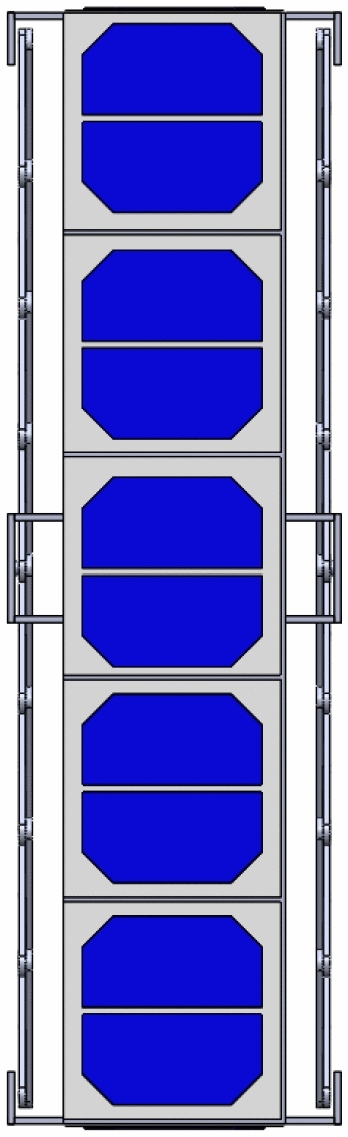
Front view of the fully extended 1U Accordion CubeSat

The Accordion design is initially equal to approximately the standard 1U CubeSat when folded, but it can extend up to 50 cm using scissors mechanism, allowing more surface area of solar cells for power generation. The fully extended CubeSat is shown in Figs. 15 and 16. This figures show the same mechanism explained in Fig. 7 with the panels added on them.

#### Power Generated by Accordion Cubesat

**Fig. 17:**
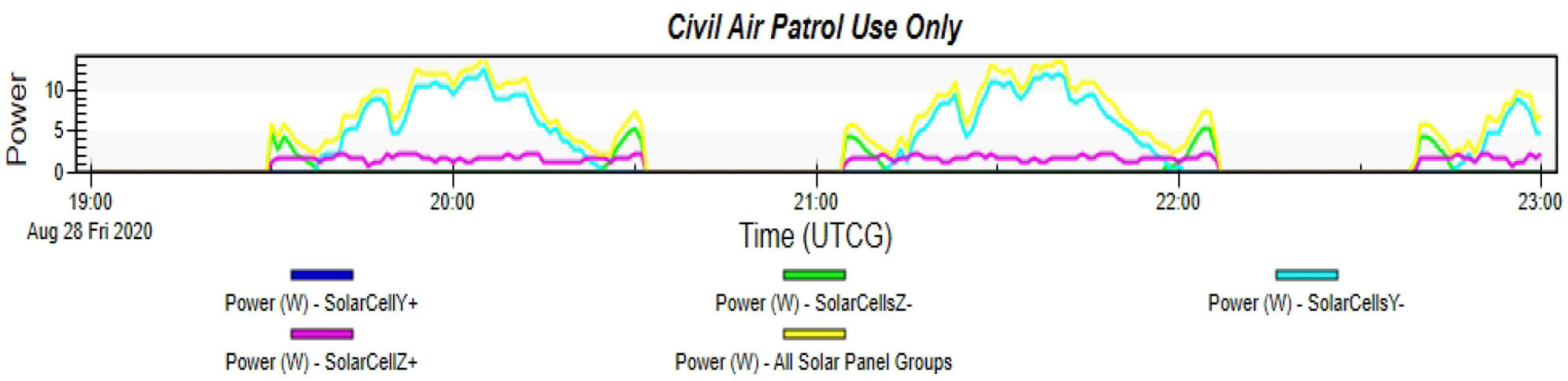
Graph of power generated by the Accordion CubeSat

Simulating the Accordion CubeSat on STK while using the orbit details mentioned in Table 2. The simulation runs for a complete day with different time intervals. This graph in Fig. 17 shows the power generated by this type of CubeSat in cells located in the Y+, Y−, Z+, and Z− of the cube individually and all combined. Therefore, the maximum power generated by the accordion CubeSat is 14.009 W, while the average power is 12.265 W. Table 3 shows the peak power generated and their timings.

**Table 3:** Power generated by Accordion CubeSat from STK

Time	Power	Solar
(UTCG)	(W)	intensity
28 Aug 2020 20:04:00.00	12.971	1.000000
28 Aug 2020 20:04:20.00	13.490	1.000000
28 Aug 2020 20:04:40.00	14.009	1.000000
28 Aug 2020 20:05:00.00	14.009	1.000000
28 Aug 2020 20:05:20.00	13.490	1.000000

#### Comparison to 1U CubeSat

**Fig. 18:**
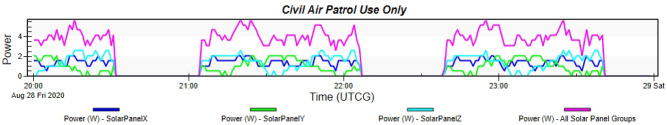
Graph of power generated by the Standard 1U Cubesat

Here is the standard 1U CubeSat placed in the same orbit and simulated for the same duration for an accurate comparison. The simulation gives the graph in Fig. 18. This graph shows the power generated by this type of CubeSat in cells located on the surfaces of the solar panel. Therefore, the maximum power generated by the Standard 1U CubeSat is 5.713 W, while the average power is 4.259 W. Table 4 shows the peak power generated and their time.

**Table 4:** Power generated by Standard 1U Cubesat from STK

Time (UTCG)	Power (W)	Solar intensity
28 Aug 2020 21:22:00.00	5.193	1.000000
28 Aug 2020 21:23:00.00	4.674	1.000000
28 Aug 2020 21:24:00.00	5.713	1.000000
28 Aug 2020 21:25:00.00	5.193	1.000000
28 Aug 2020 21:26:00.00	4.674	1.000000

### Drag-Sail CubeSat

**Fig. 19:**
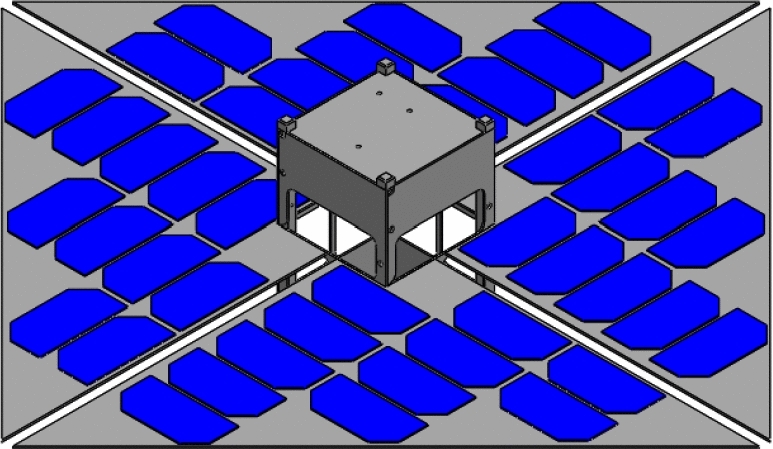
Isometric view of the fully extended 2U Drag-sail CubeSat

Meanwhile, for the Drag-Sail design, it is initially equal to the standard 2U CubeSat when folded, but it can extend up to 500 $\times$ 500 $\mathrm{{cm}}^2$ using the drag-sail mechanism, allowing more surface area of solar cells for the power generation. The fully extended CubeSat is shown in Fig. 19. This figure is the same as Fig. 19 with the panels added on it.

#### Power Generated by Drag-Sail Cubesat

**Fig. 20:**
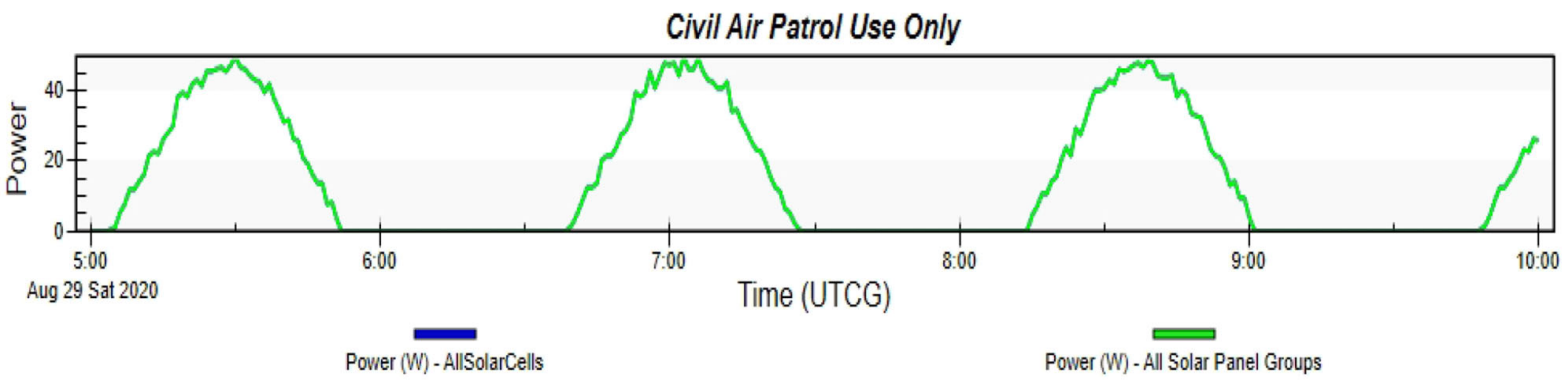
Graph of power generated by the Drag-Sail CubeSat

Simulating the Drag-Sail CubeSat on STK while using the orbit details mentioned in Table 2. The simulation runs for a complete day with different time intervals. The simulation gives the graph in Fig. 20. This graph shows the power generated by this type of CubeSat in cells located on the only surface of that solar panel. Therefore, the maximum power generated by the Drag-Sail CubeSat is 50.026 W, while the average power is 44.859 W. Table 5 shows the peak power generated and their time.

**Fig. 21:**
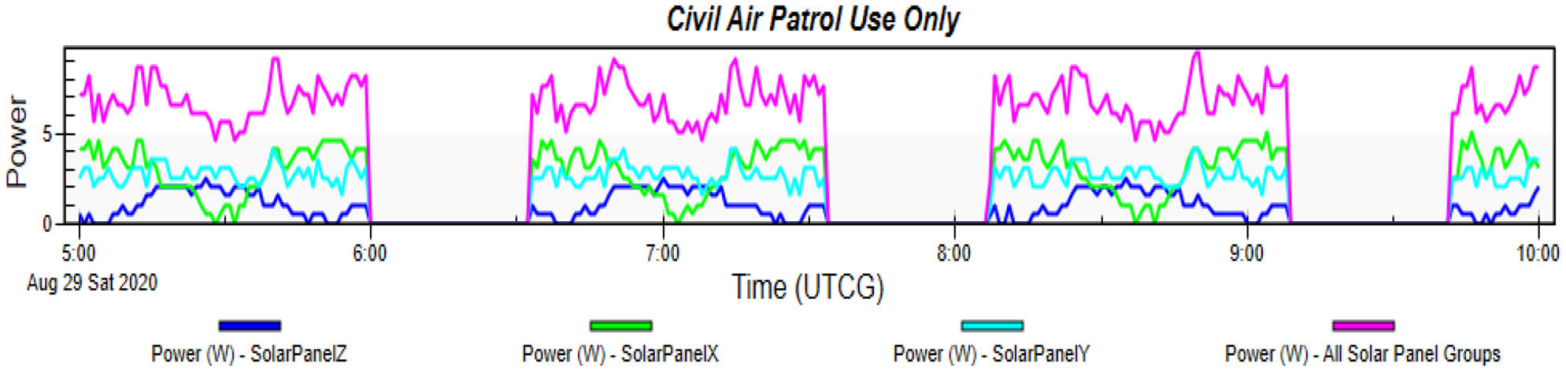
Graph of power generated by the Standard 2U Cubesat

**Table 5:** Power generated by Drag-Sail CubeSat from STK

Time (UTCG)	Power (W)	Solar intensity
28 Aug 2020 05:31:35.00	50.026	1.000000
28 Aug 2020 05:32:35.00	46.728	1.000000
28 Aug 2020 05:33:35.00	44.529	1.000000
28 Aug 2020 05:34:35.00	43.430	1.000000
28 Aug 2020 05:35:35.00	39.581	1.000000

#### Comparison to 2U CubeSat

Here is the standard 2U CubeSat placed in the same orbit and simulated for the same duration for an accurate comparison. The simulation gives the graph in Fig. 21. This graph shows the power generated by this type of CubeSat in cells located on the surfaces of the solar panel. Therefore, the maximum power generated by the Standard 2U CubeSat is 8.657 W, while the average power is 7.050 W. Table 6 shows the peak power generated and their time.

**Table 6:** Power generated by Standard 2U Cubesat from STK

Time (UTCG)	Power (W)	Solar intensity
28 Aug 2020 09:56:00.00	8.148	1.000000
28 Aug 2020 09:57:00.00	7.130	1.000000
28 Aug 2020 09:58:00.00	7.639	1.000000
28 Aug 2020 09:59:00.00	8.657	1.000000
28 Aug 2020 10:00:00.00	8.657	1.000000

### Overview of the Solar Power Generation

The STK analysis results for the solar power generation of designs 1 and 2 which are the Accordion CubeSat and Drag-Sail CubeSat are shown in Figs. 17 and 20. The maximum power generated by the Accordion CubeSat is 14.009 W, while the Drag-Sail CubeSat generates 50.026 W. Furthermore, comparing these results with the solar power generation of the standard 1U and 2U CubeSat showed in Figs. 18 and 21 which have maximum power of 5.713 W and 8.657 W, respectively. Therefore, the Accordion CubeSat performs 145.212% better than the standard 1U CubeSat. Also, the Drag-Sail CubeSat has higher power generation of 477.867% than the standard 2U CubeSat.

## Microgravity Experiment

### Safety

For the parabolic flight, there were many constraints that were taken into consideration at the designing and implementation phases. The prototype size should be less than 30 $\times$ 30 $\times$ 20 and its size should be less than 5 kg, total power consumption should be lower than 100 W. For safety at deployment, and a G-Load test must be made for prototypes before deployment.

#### G-Force for Accordion Design

**Fig. 22:**
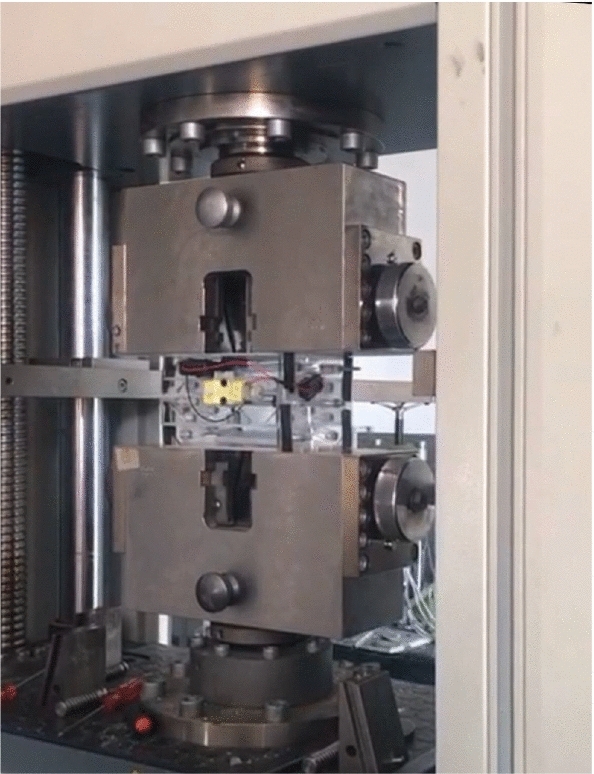
G-Force test

The G-Force for the accordion design is an essential part in the project to ensure the safety of the cube while the deployment of the microgravity experiment. Without this test, all the experiment may fail. There are many methods for these tests; some can be done using analysis tool such as ANSYS and some can done using practical laboratories. The test for the accordion design is done in the laboratory. The G-Force test for the accordion design was implemented in German University in Cairo (GUC) crash test laboratory to the grantee that the cube is safe while deployment of the flight. Figure 22 shows a sample of many tests that were implemented on the cube. The test was a success/failure test. The crash test showed that the accordion extendable CubeSat will be safe during deployment. It showed that it will not be broken from the three directional gravitational forces that it will be subjected to them at the deployment phase.

#### G-Force for Drag-Sail Design

G-Force test was performed on the drag-sail design to ensure that the cube will withstand the loading and boundary conditions during launching. The static structural analysis was done using ANSYS software [12]. This analysis estimated equivalent stresses and total deformation for all the bodies of the drag-sail structure. Aluminum alloy 6061-T6 was chosen to be the material of the 4 rails, geneva cam, and the frame of the cube which consists of 6 sides because of its relatively high yield strength, low cost, easy machinability, and lightweight. Table 7 shows the mechanical of Aluminum alloy 6061-T6 [13].

**Table 7:** Mechanical properties of Aluminum alloy 6061-T6

Property	Value and unit
Ultimate tensile strength	310 MPa
Tensile yield strength	276 MPa
Poisson’s ratio	0.33
Modulus of elasticity	68.9 GPa
Shear modulus	26 GPa
Bulk modulus	67 GPa
Density	2700 kg$/\mathrm{m}^3$

**Fig. 23:**
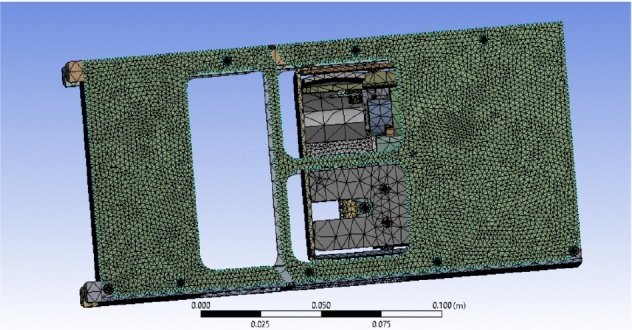
Drag-sail structure after meshing

While Steel AISI 1020 was chosen for the 32 bolts in the design and the printed circuit board (PCB) which has a tensile yield strength of 294 MPa and a tensile ultimate strength of 394 MPa, steel AISI 1035 was chosen for the motor which has a tensile yield strength of 460 MPa and a tensile ultimate strength of 460 MPa, and AL 2024 was chosen for the controller Arduino which has a tensile yield strength of 324 MPa and a tensile ultimate strength of 324 MPa. Before the analysis, meshing using tetrahedron elements was performed on the structure which consists of 665,621 elements and 1,214,592 nodes, as shown in Fig. 23.

In the quasi-static analysis, the load which consists of three vectors in the *X*-, *Y*-, and *Z*-directions and a magnitude of 2G m/s$^2$ (19.6 m/s$^2$ )in each of the three directions was applied in the geometric centroid of the structure which is located in $x=43.484 \;\mathrm{{mm}},\, y= 139.02\; \mathrm{{mm}},\, z=77.949 \;\mathrm{{mm}}$. While, the boundary conditions were presented as 16 fixed supports: 4 in the bottom of the rails, 4 in the top of the rails, and 2 on the rails in each of the rest 4 sides of the cube.

**Fig. 24:**
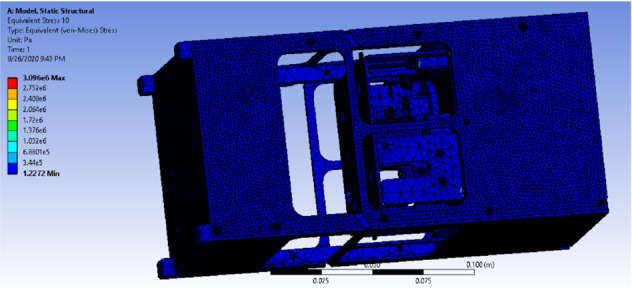
Equivalent (von-Mises) stress of the drag-sail design

Figure 24 shows the distribution of equivalent (von-Mises) stress of the drag-sail design. It shows that the maximum stress 3 MPa which guarantees the safety of the cube when it is compared with the minimum yield strength of all the materials used which is 276 MPa. This ensures that the cube is safe during deployment when it is clamped from all directions.

**Fig. 25:**
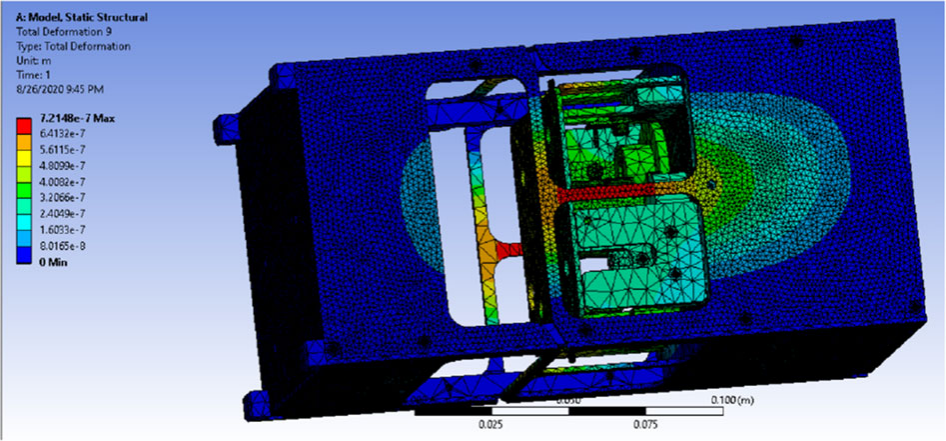
Deformation distribution of the drag-sail design

Figure 25 shows the deformation of the cube design that has the previously mentioned load and supports Where the maximum deformation is a very small value $7.2148\times 10^{-7}$ Pa which is far less compared to the dimensions of the design.

Another configuration for the fixed supports was applied where the supports were placed on the four rails in the bottom only and the force was of magnitude 2G m/s$^2$ (19.6 m/s$^2$ ) in the *X*-, *Y*-, *Z*-directions simultaneously.

**Fig. 26:**
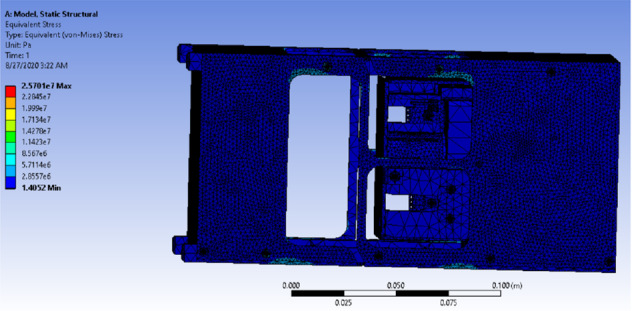
Stress distribution when the 4 corners are clamped

Figure 26 shows the maximum equivalent (von-Misses) stress when the cube is clamped from the 4 corners of the bottom. The maximum stress is 25 MPa where the minimum yield of all the materials used equals 276 MPa. This guarantees that the cube is safe during deployment when it is clamped from the bottom.

**Fig. 27:**
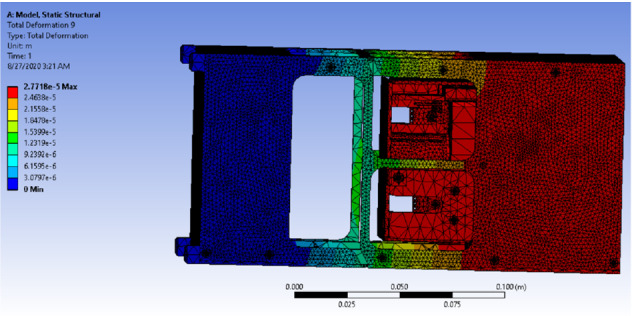
Deformation when the 4 corners are clamped

Figure 27 shows the deformation of the cube design when it’s clamped from the bottom and a 2G load affected it from *X*-, *Y*-, *Z*-directions simultaneously where the maximum deformation is a very small value $2.7718\times 10^{-5}$ Pa which is far less compared to the dimensions of the design.

Both configurations were proved to be safe for the CubeSat during launching process which means that the cube will not fail due to excess in stress or deformation.

### Falcon 20 Parabolic Flight

**Fig. 28:**
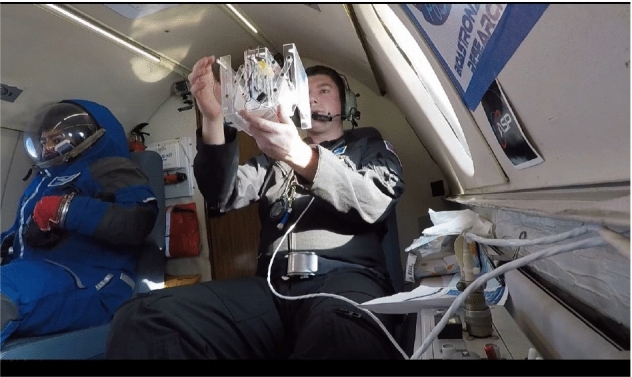
First photo of the parabolic flight experiment

**Fig. 29:**
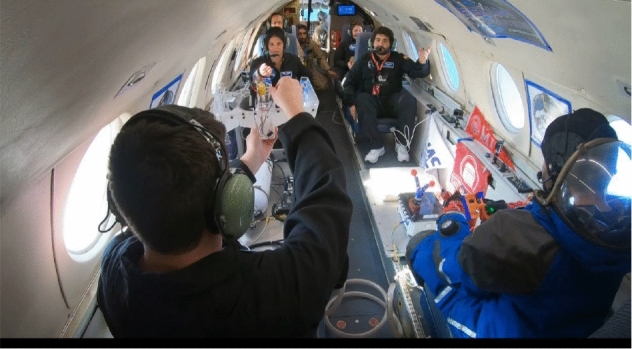
Second photo of the parabolic flight experiment

The flight includes 6 parabolas, each consisting of 20 s of reduced gravity. The flights were executed by the National Research Council (NRC) in Canada with the cooperation of Integrated Space Flight and Project PoSSUM. There was up to 3 min of level to be used for setting up experimental procedures. The material that was used for the prototype was acrylic. Laser cutting is used in the manufacturing process to convert the parts from SOLIDWORKS into a real prototype. The prototype is tested in a parabolic flight, as shown in Figs. 28 and 29. The drag-sail is not tested yet due to the COVID-19 pandemic. Figure 28 and 29 shows the experiment. By comparing with the analytical results; the cube were safe in the launching phase as proved in the analytical results, and it was stable (no extra side forces or external torques as proved in the motion analysis)

## Conclusion

This study focused on increasing the power generated by the CubeSat which increases the functionality and processing capabilities, so it can be used in more challenging missions. Two expandable designs are proposed which are accordion CubeSat (1U) and drag-sail CubeSat (2U). Different analyses are applied to both CubeSats such as structural, dynamical, and power analysis. The accordion CubeSat is implemented and tested in the microgravity. Results show that both designs mechanism has neither side force nor extra torque that guarantees the cube stability in space. The generated power efficiency by the proposed CubeSats outperformed the standards, where the accordion CubeSat exceeded the standard 1U by 145.212%, while the drag-sail CubeSat exceeded the standard 2U by 477.867%. The microgravity experiment is done in a reduced-gravity aircraft during a parabolic flight and it shows that the accordion design is stable and can be used for future CubeSats. Future research directions include manufacturing and testing the drag-sail CubeSat to ensure its safety during flights and to be tested in microgravity, measuring the produced vibrations during the deployment of both CubeSats. Also, consider different orbital scenarios for the satellites.
